# Draft Genome Sequence of the Mercury-Resistant Strain Acinetobacter baumannii I43

**DOI:** 10.1128/genomeA.00283-18

**Published:** 2018-04-12

**Authors:** Kátia Aparecida de Siqueira, Ivani Souza Mello, William Pietro-Souza, Tiago Antônio de Oliveira Mendes, Marcos Antônio Soares

**Affiliations:** a Department of Botany and Ecology, Laboratory of Biotechnology and Microbial Ecology, Institute of Biosciences, Federal University of Mato Grosso, Cuiaba, Mato Grosso, Brazil; b Department of Biochemistry and Molecular Biology, Federal University of Viçosa, Viçosa, Minas Gerais, Brazil

## Abstract

Here, we report the draft genome sequence of the Acinetobacter baumannii strain I43, which is highly resistant to mercury. The Illumina-based sequence analysis revealed a genome of approximately 4,520,353 bp composed of 4,091 coding sequences.

## GENOME ANNOUNCEMENT

Acinetobacter baumannii I43 is a mercury-resistant (MIC > 250 µg/ml HgCl_2_) endophytic bacterial strain isolated from the roots of Aeschynomene fluminensis samples collected in the main continental wetlands of Brazil; it promotes the growth of host plants contaminated with mercury (data not shown). Here, we report the annotated genome of this mercury-resistant species.

Sample DNA was extracted using a PowerSoil DNA isolation kit (Mo Bio, USA), and the libraries were prepared using a Nextera DNA library preparation kit (Illumina, USA). After quantification by quantitative PCR (qPCR) and size quality control of the libraries using a 1200 Bioanalyzer (Agilent, USA), the DNA sample was sequenced using the HiSeq4000 sequencer (Illumina, USA) in the 150-bp paired-end mode. Adapters and low-quality sequences were removed using Trimmomatic software (1).

The whole-genome sequence was *de novo* assembled using SPAdes (2), resulting in 1,070 scaffolds with a total size of 4,520,353 bp, a GC content of 40%, an *N*_50_ value of 197,309 bp, and a mean read length of 584,768 bp. Genome completeness analysis with BUSCO (3) showed results of 96.4% complete (0.4% duplicated), 0.4% fragmented, and 3.2% missed, as well as the presence of 452 genes, indicating that the assembled genome covered most of the coding regions.

Annotation of the genome sequence using the Rapid Annotations using Subsystems Technology (RAST) server (4) and antiSMASH (5), followed by tRNA and rRNA detection using ARAGORN (6) and RNAmmer (7), respectively, identified 4,091 coding sequences, 66 tRNAs, 9 rRNAs, and the presence of resistance genes from the *mer* system (*merA*, *merB*, *merC*, *merD*, *merE*, *merP*, and *merT*), which are important genes for mercury detoxification. A preliminary analysis using antiSMASH resulted in 7, 8, and 13 gene clusters related to lasso peptide synthesis, arylpolyenes, and nonribosomal peptide synthetases, respectively. Altogether, our findings reveal that the genome of A. baumannii strain I43 encodes molecules that can be potential candidates for the development of new products of biotechnological interest.

### Accession number(s).

The complete genome sequence of A. baumannii I43 was deposited in GenBank under the accession number NXDV00000000. The version described in this paper is the first version, NXDV01000000.
